# The variations of *IL-23R* are associated with susceptibility and severe clinical forms of pulmonary tuberculosis in Chinese Uygurs

**DOI:** 10.1186/s12879-015-1284-2

**Published:** 2015-12-01

**Authors:** Daobin Jiang, Atikaimu Wubuli, Xin Hu, Syed Ikramullah, Abudoujilili Maimaiti, Wenbao Zhang, Qimanguli Wushouer

**Affiliations:** Department of Pulmonology, The First Affiliated Hospital of Xinjiang Medical University, Urumqi, China; Department of Pulmonology, Xinjiang Uygur Autonomous Region Hospital of Traditional Chinese Medicine, Urumqi, Xinjiang 830054 China; Xinjiang Uygur Autonomous Region Respiratory Physiology Pathology Key Laboratory, Urumqi, Xinjiang 830054 China; Clinical Medical Research Institute, The First Affiliated Hospital of Xinjiang Medical University, Urumqi, Xinjiang 830054 China

**Keywords:** IL23 receptor, Tuberculosis, Susceptibility, SNP, Copy number variation, Drug-resistant, Cavitary lesion

## Abstract

**Background:**

The incidence of tuberculosis (TB) remains high among Chinese Uygurs (a long-dwelling ethnic minority in Xinjiang) in China and the variants in *IL-23R* likely contribute to individual’s diversity in host response during infection.

**Methods:**

A hospital based one to one matched case–control study was performed to assess the role of single nucleotide polymorphisms (SNPs) and copy number variation (CNV) of *IL-23R* in susceptibility and clinical features of pulmonary TB in Chinese Uygurs. Thirteen SNPs in *IL-23R* were genotyped by multiplex SNaPshot and a CNV was analyzed using Taqman real-time PCR in 250 pairs of pulmonary TB patients and controls.

**Results:**

The SNP rs7518660 (OR = 4.78, 95 % CI 3.14–8.52) and the CNV in *IL23R* (OR = 2.75, 95 % CI 1.51–4.98) were significantly associated with susceptibility to pulmonary TB. The SNP rs11465802 (OR = 3.23, 95 % CI 1.85–5.62) was significantly associated with drug-resistance and the SNP rs1884444 (OR = 3.61, 95 % CI 1.90–6.85) was significantly related to cavitary lesion in Chinese Uygurs.

**Conclusions:**

Our study shows for the first time that SNP and CNV in *IL23R* were associated with susceptibility, drug resistance and cavity formation of pulmonary TB. Our findings indicate that these *IL-23R* polymorphisms may be considered as risk factors for active pulmonary TB and its severe clinical forms.

**Electronic supplementary material:**

The online version of this article (doi:10.1186/s12879-015-1284-2) contains supplementary material, which is available to authorized users.

## Background

China has the world’s second largest tuberculosis (TB) burden, accounting for 12 % of the global total cases in 2012 [1], and the Xinjiang Uygur Autonous Region (Xinjiang) in northwestern China, has one of the highest rates of incidence and mortality of TB in China [2–4]. The Chinese Uygur, one of the minority ethnic groups, has higher prevalence of TB than the Chinese Han and other Chinese minorities in Xinjiang, China [3], indicating that this group of people may be susceptible to TB.

The essential role of human genetic factors in susceptibility to TB has been confirmed by twin studies [5], linkage analysis [6], candidate gene association analysis (reviewed in [7]) and genome-wide association studies (GWAS) [8]. However, cumulative evidence suggests that there is an ethnic difference in the polymorphisms of candidate genes associated with TB susceptibility [9, 10]. Thus, identification of variants of the genes linked with TB susceptibility is still crucial for understanding both the mechanism underlying TB susceptibility and gene function.

IL-23, a member of the IL-12 cytokine family, is a key proinflammatory cytokine in innate and adaptive immune systems. This cytokine plays a pivotal role in the differentiation of the native CD4^+^ T cells into Th17 [11]. It has been shown that IL-17, mostly produced by Th17 cells, plays a crucial role in control of *M. tuberculosis* infection [12]. Furthermore, IL-23 is required for long-term control of *M. tuberculosis* [13]. The activity of IL-23 is mediated by its binding to IL23R complex, which is composed of IL-12Rβ1 and IL-23R [14]. Recent studies have shown that IL23R might also affect other dependent cytokines, such as IL-1, IL-6 and tumor necrosis factor (TNF) [15]. These cytokines are also critical for the progression of tuberculosis [7, 16]. However, there has been only one study showing one single nucleotide polymorphism (SNP) in *IL-23R* was associated with susceptibility to TB [17]. In addition, *IL23R* exhibits a copy number variation (CNV) [18, 19], which could contribute to individual’s diversity in host response during infection and inflammation, and is possibly associated with TB.

A hospital based one to one matched case–control study was conducted to determine whether the SNPs and CNV of *IL23R* are associated with susceptibility and clinical forms of pulmonary TB in Chinese Uygurs.

## Methods

### Study population

All participants were from Kashgar Prefecture, Xinjiang, China. The area was selected due to the high incidence of TB as well as the uniform ethnicity (with common genetic background of Chinese Uygur [20]) and socioeconomic status. The ethnicity recording of all the participants as Chinese Uygur were derived from their self-declaration and checked by their identity (ID) card. 250 unrelated Uygur patients diagnosed with active pulmonary TB at Kashgar Prefecture Chest Hospital were enrolled from October 2013 to April 2014. Criteria for inclusion as pulmonary TB cases were: 1) positive sputum culture; 2) age range 18-75 years; 3) excluding those with comorbidities such as lung carcinoma, asthma, bronchiectasis, diabetes mellitus and other immunosuppressive conditions. Patients meeting all the criteria were recruited. An equal number of age- (within 3 years) and sex-matched Uygur healthy controls from the Health Checkup Center of Kashgar Prefecture People’s Hospital were enrolled during the same period. Those controls were negative both for history of TB and T. SPOT. TB assay. All participants had *BCG* vaccination and were HIV seronegative.

The protocol was approved by the Ethics Committee of The First Affiliated Hospital of Xinjiang Medical University, Urumqi, China. Written informed consents were obtained from all participants.

Clinical features of pulmonary TB including drug resistance of the strain, elapsed time to sputum culture conversion, pulmonary infiltration and cavitary lesion were reviewed as well as age, gender, body measure index (BMI), smoking status and education background possibly to be risk factors for developing pulmonary TB. The culture, identification and drug sensitivity test of *M. tuberculosis* strains were performed according to the national criteria in China [21]. Four first-line anti-TB drugs (isoniazid, rifampicin, ethambutol, and streptomycin) and seven second-line anti-TB drugs (Paminosalicylicacid, Ofloxacin, Capreomycin, Amikacin, Kanamycin, Ethionamide, and Cycloserine) were adopted to detect drug resistance, as described elsewhere [22]. Drug sensitivity was defined as sensitivity to all the tested drugs and drug resistance as resistance to at least one tested drug, as described elsewhere [23]. Pulmonary infiltration and cavity were evaluated on the basis of chest CT scanning obtained at the beginning of the treatment, which was read by independent physician not affiliated with the study. Pulmonary infiltration on chest CT scanning was divided into two categories, one involving one third or less of one lung, and the other involving more than that. The commercially available T. SPOT. TB assay (Oxford Immunotec, Abingdon, UK) was performed to exclude latent *M. tuberculosis* infection (LTBI) in controls, due to some candidate genes being related to TB as well as LTBI [24].

### Genomic DNA extraction and marker selection

Genomic DNA was extracted from peripheral blood mononuclear cells (PBMCs) of the samples with the TIANamp blood Genomic DNA kit (Tiangen, Beijing, China).

To confirm SNPs in Chinese Uygurs, we initially sequenced all 11 exons of *IL-23R* using ABI 3730 DNA Analyzer (Additional file 1: Figure S1A) in 10 pairs of samples, with each exon flanked with partial intronic sequences. The primers were designed by Primer 5.0 software (Additional file 2: Table S1). Thirteen common SNPs were identified in those regions and all were selected for further genotyping (Additional file 3: Table S2). The CNV in exon 11 of *IL23R* demonstrated previously [19] was selected for further analysis.

### SNP genotyping and copy number measurement

SNP genotyping was performed by the multiplex SNaPshot technique (Additional file 1: Figure S1B). In brief, the primers and probes were designed by Primer 5.0 software (Additional file 4: Table S3). Multiplex PCR products amplified by HotStarTaq (Qiagen) were purified with shrimp alkaline phosphatase (SAP) (Promega, Madison, USA) and Exonuclease I (*Exo*I) (Epicentre, Madison, USA). The purified Multiplex PCR products were then used as templates for a minisequencing extension reaction using the SNaPshot Multiplex kit (Applied Biosystems, Carlsbad, CA, United States). The SNaPshot PCR products were also purified by SAP, and then detected by capillary electrophoresis on an ABI 3130XL DNA Analyzer (Applied Biosystems). The call rate per SNP exceeded 98 %. The CNV in *IL-23R* of all participants was determined by Taqman real-time PCR, as described elsewhere [19]. The call rate for copy number measurement reached 100 %. Furthermore, Accucopy assay [25] and droplet digital PCR (ddPCR) [26] were used to validate the copy number of 25 pairs randomly selected from those tested samples (Additional file 5: Figure S2 and Additional file 6: Figure S3), which account for 10 % of the total samples.

### Statistical analysis

Continuous variables were compared using the Student’s *t* test. Hardy–Weinberg equilibrium (HWE) was assessed for all SNPs by *χ*^2^ goodness of fit test or Fisher’s exact test. SNPs that deviated from the HWE (*P* < 0.05) or had frequencies of variants of < 5 % in the control group were excluded. Linkage disequilibrium blocks were structured and haplotype frequencies were calculated using Haploview version 4.2 (Daly Lab at the Broad Institute Cambridge, MA, United States). The Linkage Disequilibrium (LD) between SNPs and CNV was determined both at the genotype and haplotype levels in the control group, also using Haploview version 4.2, as described elsewhere [27]. The associations between pulmonary TB and *Il-23R* polymorphisms were calculated by conditional logistic regression analysis, for three different genetic models (dominant, recessive and additive), then adjusted by covariants for all analyses. The association of each polymorphism with pulmonary TB clinical forms was confirmed by unconditional logistic regression. Bonferroni correction was applied for multiple testing. All *P* values reported were two-tailed, and a level of 0.05 was considered statistically significant. Statistical analysis was performed using SAS version 9.2 (SAS institute, Cary, NC, United States).

## Results

This study consisted of 250 pulmonary TB cases and 250 controls, matched with age and sex. Males accounted for 55.6 % and females 44.4 % in each group. The age was 44.34 ± 14.17 years for cases and 47.34 ± 16.20 years for controls. Among those 250 pulmonary TB patients, there were 172 drug-sensitive patients and 78 drug-resistant ones. In those 78 drug-resistant pulmonary TB patients, there were 10 multidrug-resistant tuberculosis (MDR). There was no significant difference in age, gender, smoking, alcohol intake, presence of TB history of houshold or education background between pulmonary TB patients and controls. As expected, BMI showed significant difference between these two groups, demonstrating that thin individuals (BMI < 18.5) (OR = 3.93, 95 % CI 2.71–5.71) are more likely to suffer from pulmonary TB in Chinese Uygurs (Table 1).

**Table 1:** Basic characteristics of the participants in the study

Characteristics	Controls (*N* = 250)	PTB Cases (*N* = 250)	OR (95 % CI)
Age, year			
Mean ± SD	47.34 ± 16.20	44.34 ± 14.17	
Gender			
Male : Female	139:111	139:111	
BMI			
<18.5 : ≥18.5	92:158	174:76	3.93 (2.71–5.71)
Smoking status			
Smoker : No-smoker	9:241	5:245	0.55 (0.18–1.65)
Alcohol intake status			
Drinker : No-drinker	12:238	8:242	0.66 (0.26–1.63)
Presence of TB history of houshold			
Presence : Absence	24:226	36:224	1.51 (0.87–2.62)
Education background			
Junior high school or below : Senior high school or above	116:134	124:126	1.14 (0.80–1.62)

*PTB* pulmonary tuberculosis, *OR* odds ratio, *CI* confidence interval, *SD* standard deviation, *BMI* body measure index

The ethnicity recording of the study population as Chinese Uygur were derived from their self-declaration and checked by their identity (ID) card

### Association of the *IL-23R* genotype and allele frequencies with pulmonary tuberculosis

Genotype frequencies for 9 SNPs (rs1884444, rs11465770, rs10889664, rs11465788, rs7518660, rs11465802, rs11465804, rs11209016 and rs10889677) included were in HWE in both case and control groups (*P* > 0.05), whereas other 4 SNPs (rs6687620, rs2863212, rs7530511 and rs10889671) were excluded, due to their deviation from HWE (*P* < 0.05) in the control group. Of the 9 SNPs included initially, 3 SNPs (rs11465770, rs11465804 and rs11209026) were also excluded, because of their frequencies of variants of < 5 % in the controls. Finally, 6 SNPs (rs1884444, rs10889664, rs11465788, rs7518660, rs11465802 and rs10889677) were included for further analysis.

**Fig. 1:**
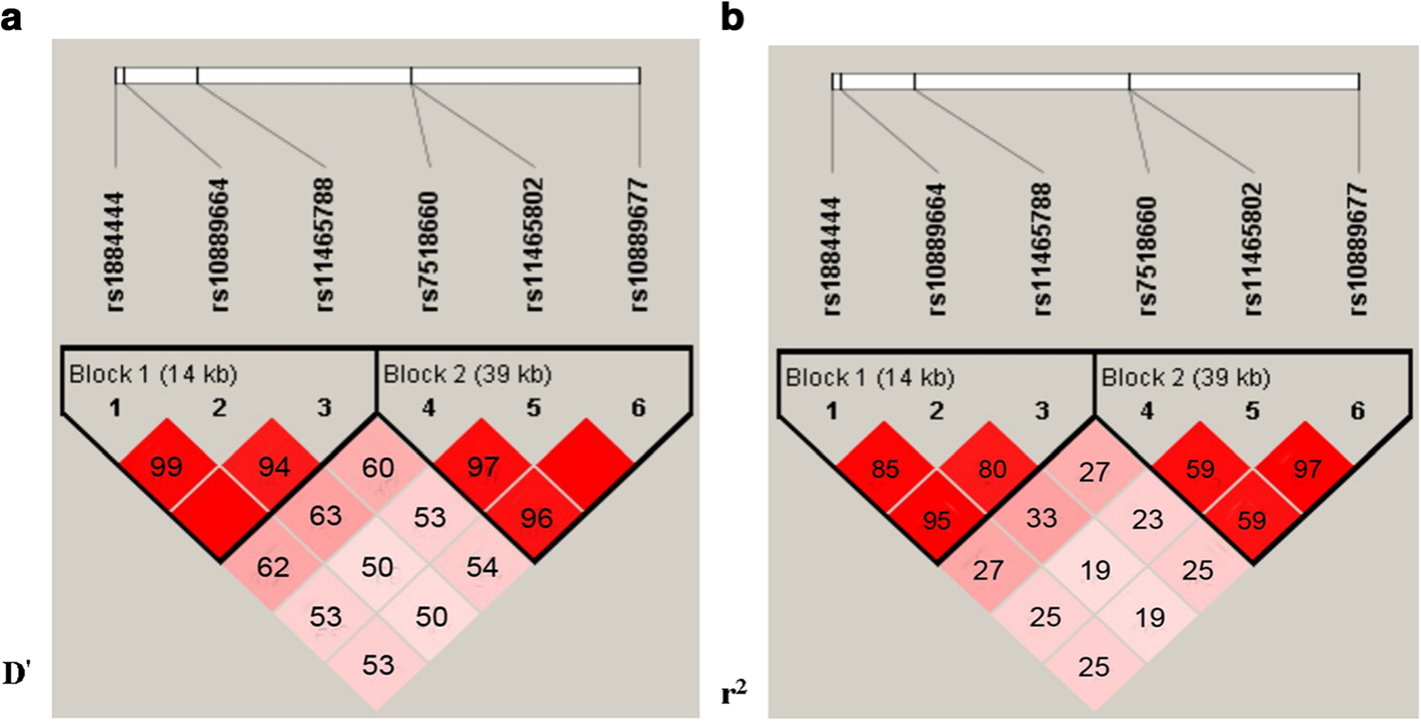
Linkage Disequilibrium Plot of SNPs of IL-23R in the control group. Linkage disequilibrium (LD) blocks in the IL23R polymorphisms black squares indicate significant allelic association between groups of SNPs from the control subjects measured by the D' (*left panels*) and r^2^ (*right panels*) statistics. A D' or r^2^ cutoff of 0.8 is used for LD. High D' values are dark, low D' values are light

The SNP rs7518660 (AA vs GG, OR = 12.73, 95 % CI 7.16–22.6; AG vs GG, OR = 2.99, 95 % CI 1.87–4.79; AA + AG vs GG, OR = 4.78, 95 % CI 3.14–8.52) showed significant association with pulmonary TB susceptibility (Table 2, Table 3). The genotypes distribution frequencies of rs10889677 (CC vs AA OR = 1.85, 95 % CI 1.13–3.05) showed significant difference between the PTB cases and controls (Table 2). However, after adjusting for the covariant of BMI, there was no association between rs10889677 (CC + AC vs AA OR = 0.88, 95 % CI 0.42–1.23) and pulmonary TB (Table 3). There was moderate to high LD in the control group between these 2 SNPs (r^2^ = 0.59, D' = 0.96). There was also moderate to high LD in controls between rs7518660 and rs11465802 (r^2^ = 0.59, D' = 0.97), and high LD between rs10889677 and rs11465802 (D' = 0.97) (Fig. 1). Unexpectedly, there was no association between rs11465802 and susceptibility to pulmonary TB in Chinese Uygurs. The alleles of rs7518660 (OR = 3.56, 95 % CI 2.74–4.62) and rs10889677 (OR = 1.37, 95 % CI 1.07–1.76) also showed significant difference between the cases and the controls (Table 2).

**Table 2:** Genotype and allele frequencies distribution of *IL-23R* polymorphisms between controls and cases

dbSNP ID/CNV	Genotype/Allele	Controls n (%)	PTB cases n (%)	OR (95 % CI)
rs1884444	TT	82 (33.1)	87 (34.8)	1
	GT	122 (49.2)	114 (45.6)	0.88 (0.59–1.31)
	GG	44 (17.7)	49 (19.6)	1.05 (0.63–1.74)
	T	286 (57.7)	288 (57.6)	1
	G	210 (42.3)	212 (42.4)	1.00 (0.78–1.29)
rs10889664	CC	93 (37.5)	103 (41.2)	1
	CT	117 (47.2)	109 (43.6)	0.84 (0.57–1.23)
	TT	38 (15.3)	38 (15.2)	0.90 (0.53–1.53)
	C	303 (61.1)	315 (63.0)	1
	T	193 (38.9)	185 (37.0)	0.92 (0.71–1.19)
rs11465788	TT	85 (34.7)	87 (34.9)	1
	CT	120 (49.0)	114 (45.8)	0.93 (0.63–1.38)
	CC	40 (16.3)	48 (19.3)	1.17 (0.70–1.96)
	T	290 (59.2)	288 (57.8)	1
	C	200 (40.8)	210 (42.2)	1.06 (0.82–1.36)
rs7518660	GG	105 (42.3)	33 (13.2)	1
	AG	116 (46.8)	109 (43.6)	2.99 (1.87–4.79)
	AA	27 (10.9)	108 (43.2)	12.73 (7.16–22.6)
	G	326 (65.7)	175 (35.0)	1
	A	170 (34.3)	325 (65.0)	3.56 (2.74–4.62)
rs11465802	CC	74 (30.0)	70 (28.0)	1
	AC	121 (49.0)	121 (48.4)	1.06 (0.70–1.60)
	AA	52 (21.0)	59 (23.6)	1.20 (0.73–1.97)
	C	269 (54.5)	261 (52.2)	1
	A	225 (45.5)	239 (47.8)	1.10 (0.85–1.41)
rs10889677	AA	76 (30.9)	57 (22.9)	1
	AC	119 (48.4)	121 (48.6)	1.36 (0.89–2.08)
	CC	51 (20.7)	71 (28.5)	1.85 (1.13–3.05)
	A	271 (55.1)	235 (47.2)	1
	C	221 (44.9)	263 (52.8)	1.37 (1.07–1.76)
CNV	CN < 2	3 (1.2)	7 (2.8)	2.66 (0.68–10.41)
	CN = 2	230 (92.0)	202 (80.8)	1
	CN > 2	17 (6.8)	41 (16.4)	2.75 (1.51–4.98)

*PTB* pulmonary tuberculosis, *OR* odds ratio, *CI* confidence interval, *CNV* copy number variation, *CN* copy number

**Table 3:** Association of *IL-23R* SNPs genotypes with pulmonary TB under different genotype models

dbSNP ID	Allele	Genetic Model	OR (95 % CI)	ORc (95 % CI)	ORa (95 % CI)
rs1884444	T > G	Dominant	1.08 (0.75–1.56)	1.12 (0.83–1.77)	1.22 (0.89–1.83)
		Recessive	1.17 (0.72–1.79)	1.21 (0.77–1.84)	1.33 (0.79–1.94)
		Additive	0.86 (0.61–1.23)	0.91 (0.81–1.34)	0.93 (0.85–1.46)
rs10889664	C > T	Dominant	1.16 (0.81–1.67)	1.22 (0.90–1.83)	1.31 (0.94–1.92)
		Recessive	0.86 (0.62–1.65)	0.89 (0.72–1.88)	0.95 (0.76–1.97)
		Additive	1.16 (0.81–1.64)	1.34 (0.93–1.77)	1.54 (0.95–1.83)
rs11465788	T > C	Dominant	0.99 (0.68–1.43)	1.02 (0.74–1.65)	1.23 (0.82–1.75)
		Recessive	1.22 (0.77–1.30)	1.24 (0.82–1.43)	1.37 (0.85–1.55)
		Additive	0.88 (0.62–1.25)	0.93 (0.81–1.38)	0.95 (0.86–1.43)
rs7518660	G > A	Dominant	4.83 (3.10–7.53)	4.91 (3.25–8.17)	4.78 (3.14–8.52)
		Recessive	6.25 (3.85–10.00)	6.54 (3.62–10.34)	6.38 (3.51–10.46)
		Additive	0.88 (0.62–1.25)	0.91 (0.58–1.36)	0.93 (0.65–1.47)
rs11465802	C > A	Dominant	0.91 (0.62–1.33)	0.95 (0.57–1.46)	1.01 (0.48–1.52)
		Recessive	1.16 (0.60–1.75)	1.23 (0.54–1.86)	1.45 (0.76–1.92)
		Additive	1.02 (0.72–1.46)	1.14 (0.65–1.57)	1.26 (0.63–172))
rs10889677	A > C	Dominant	0.66 (0.44–0.99)	0.72 (0.39–1.12)	0.88 (0.42–1.23)
		Recessive	1.53 (1.01–2.31)	1.61 (0.99–1.98)	1.75 (1.05–2.03)
		Additive	1.01 (0.71–1.43)	1.15 (0.68–1.74)	1.24 (0.73–1.82)

*OR* odds ratio, *CI* confidence interval

*OR* for comparison of genetic models by conditioned logistic regression analysis

ORc = OR value after Bonferroni correction

ORa = OR value adjusted for BMI

### Association of *IL-23R* CNV with pulmonary TB

We initially sequenced 11 exons of *IL23R* from 10 pair of samples. However, we did not find the CNV reported previously [19] in those subjects. Given the much lower frequency distribution of CNV compared to SNPs [28] and the small sample size we selected, the negative result was reasonable. Taking this into consideration, we still further identified the CNV using Taqman real-time PRC in 250 pairs of samples.

**Fig. 2:**
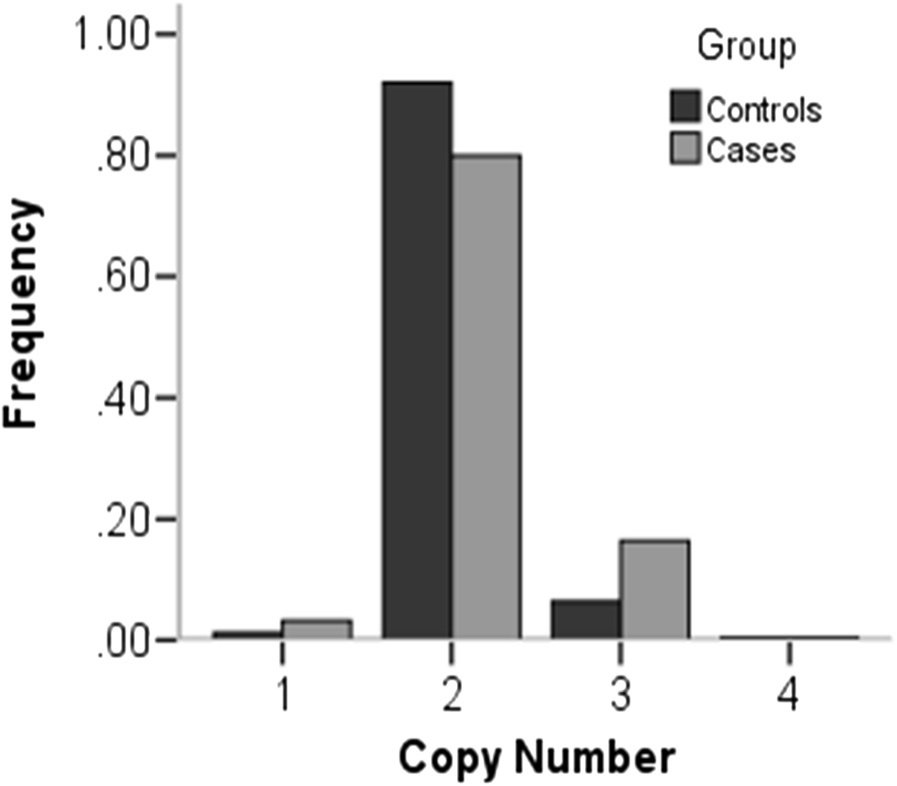
Copy number frequencies distribution of IL-23R between controls and pulmonary TB patients

Delta Ct value of *IL23R* for each sample was calculated by normalizing with the Ct value of *RnaseP* (Additional file 1: Figure S1C). Copy number was then transferred from the Delta Ct value. The copy number frequency distribution of *IL-23R* showed a significant difference (*P* = 0.001) between controls and cases (Fig. 2). The further analysis showed that the frequencies of copy number increase (OR = 2.75, 95 % CI 1.51–4.98) in patients were significantly higher than those in controls (Table 2).

It is important to determine the degree of LD between the CNV of *IL-23R* and rs7518660 and rs10889677, given that these variants were all associated with susceptibility to pulmonary TB in Chinese Uygurs. LD between the CNV and these two SNPs was determined in the control group at the haplotype level, using estimated phased as well as genotype level. There was weak LD between the CNV and rs7518660 and rs10889677 in the control group, as measured by r^2^ (r^2^ < 0.01), which was not affected by the MAF of the haplotypes (Additional file 7: Table S4).

We then used AccuCopy assay and ddPCR to validate the copy number of *IL-23R* calculated by Taqman real-time PCR with 25 pairs randomly selected from those 500 tested samples (Additional file 5: Figure S2 and Additional file 6: Figure S3). The results both showed 98 % concordant. Due to the relatively small sample size, we then calculated the power of the test. It is over 0.8, meaning that the result is reliable.

### Association of *IL-23R* haplotypes with pulmonary TB

Pairwise LD between the 6 SNPs of *IL-23R* was calculated by D' and r^2^ from controls (Fig. 1). Based on the LD, 2 block haplotypes were constructed using Haploview version 4.2. The common haplotypes (frequency > 3 %) in each block accounted for 97.3 % and 96.4 % for the cases and 100 % and 97.5 % for the controls. We found 5 haplotypes (GTC and GCC in Block1; GAA, ACA and GAC in Block2) were significantly associated (OR = 6.10, 95 % CI 4.99–7.47; OR = 6.50, 95 % CI 4.36–9.71; OR = 1.97, 95 % CI 1.57–2.47; OR = 0.61, 95 % CI 0.44–0.85; OR = 6.76, 95 % CI 4.76–9.60, respectively) with pulmonary TB (Table 4). Among these 5 haplotypes, ACA in Block2 was a protective factor for Pulmonary TB, while other 4 haplotypes were risk factors.

**Table 4:** Association between haplotypes frequencies of *IL-23R* and pulmonary TB susceptibility

Block	Haplotypes	Controls (%)	PTB Cases (%)	OR (95 % CI)
Block1				
rs1884444 rs10889664 rs11465788	TCT	70.8	27.5	1
	GTC	25.4	60.2	6.10 (4.99–7.47)
	GCC	3.8	9.6	6.50 (4.36–9.71)
Block2				
rs7518660 rs11465802 rs10889677	ACC	44.6	30.4	1
	GAA	22.8	30.6	1.97 (1.57–2.47)
	ACA	14.2	5.9	0.61 (0.44–0.85)
	AAA	11.3	8.3	1.08 (0.78–1.48)
	GAC	4.6	21.2	6.76 (4.76–9.60)

*PTB* pulmonary tuberculosis, *OR* odds ratio, *CI* confidence interval

### Association of *IL-23R* polymorphisms with clinical forms of pulmonary TB

The SNP rs11465802 (AA + AC vs CC, OR = 3.23, 95 % CI 1.85–5.62) showed significant association with drug resistance of the strain and the SNP rs1884444 (GG + GT vs TT, OR = 3.61, 95 % CI 1.90–6.85) demonstrated significant association with cavitary formation on chest CT scanning in Pulmonary TB patients. There were no significant association between the polymorphisms of *IL-23R* and conversion of sputum culture and pulmonary infiltration in the pulmonary TB patients (Table 5, Table 6).

**Table 5:** Association of *IL-23R* polymorphisms and drug-sensitivity and conversion of sputum culture in pulmonary TB patients

dbSNP ID/	Genotype	Drug-sensitivity (*N* = 250)		OR (95 % CI)	Months of treatment for Negative culture (*N* =250)		OR (95 % CI)
CNV		Drug-sensitive	Drug-resistant		≤2 months	> 2 months	
		*N* = 172 n (%)	*N* = 78 n (%)		*N* = 195 n (%)	*N* = 55 n (%)	
rs1884444	TT	72 (41.9)	31 (39.7)	1	79 (40.5)	24 (43.6)	1
	GG + GT	100 (58.1)	47 (60.3)	1.09 (0.63–1.88)	116 (59.5)	31 (56.4)	0.88 (0.48–1.61)
rs10889664	CC	85 (49.4)	38 (48.7)	1	96 (49.2)	27 (49.1)	1
	TT + CT	87 (50.6)	40 (51.3)	1.03 (0.60–1.76)	99 (50.8)	28 (50.9)	1.01 (0.55–1.83)
rs11465788	TT	73 (42.1)	32 (41.0)	1	83 (42.6)	22 (40.0)	1
	CC + CT	99 (57.9)	46 (59.0)	1.06 (0.62–1.83)	112 (57.4)	33 (60.0)	1.11 (0.60–2.05)
rs7518660	GG	8 (4.7)	4 (5.1)	1	9 (4.6)	3 (5.5)	1
	AA + AG	164 (95.3)	74 (94.9)	0.90 (0.29–3.09)	186 (95.4)	52 (94.5)	0.84 (0.22–3.21)
rs11465802	CC	117 (32.0)	31 (60.3)	1	116 (59.5)	32 (58.2)	1
	AA + AC	55 (68.0)	47 (39.7)	3.23 (1.85–5.62)	79 (40.5)	23 (41.8)	1.06 (0.58–1.94)
rs10889677	AA	37 (21.5)	16 (20.5)	1	41 (21.0)	12 (21.8)	1
	CC + AC	135 (78.5)	62 (79.5)	1.06 (0.55–2.05)	154 (79.0)	43 (78.2)	0.95 (0.46–1.97)
CNV	CN = 2	138 (80.2)	62 (79.5)	1	157 (80.5)	43 (78.2)	1
	CN = 1, 3, 4	34 (19.8)	16 (20.5)	1.05 (0.54–2.04)	38 (19.5)	12 (21.8)	1.15 (0.56–2.40)

*OR* odds ratio, *CI* confidence interval

**Table 6:** Association of *IL-23R* polymorphisms and pulmonary infiltration and cavitary formation in pulmonary TB patients

dbSNP ID/	Genotype	Cavitary formation (*N* = 250)		OR (95 % CI)	Pulmonary infiltration (*N* = 250)		OR (95 % CI)
		No Cavity lesion	Cavity lesion		≤ 1/3 of one lung	>1/3 of one lung	
		*N* = 179 n (%)	*N* = 71 n (%)		*N* = 138 n (%)	*N* = 112 n (%)	
rs1884444	TT	88 (49.2)	15 (21.1)	1	58 (42.0)	45 (40.2)	1
	GG + GT	91 (50.8)	56 (78.9)	3.61 (1.90–6.85)	80 (58.0)	67 (59.8)	1.08 (0.65–1.79)
rs10889664	CC	87 (48.6)	36 (50.7)	1	68 (49.3)	55 (49.1)	1
	TT + CT	92 (51.4)	35 (49.3)	0.92 (0.53–1.59)	70 (50.7)	57 (50.9)	1.01 (0.61–1.66)
rs11465788	TT	74 (41.3)	31 (43.7)	1	57 (41.3)	48 (42.9)	1
	CC + CT	105 (58.7)	40 (56.3)	0.91 (0.52–1.59)	81 (58.7)	64 (57.1)	0.94 (0.57–1.55)
rs7518660	GG	7 (4.1)	5 (7.0)	1	7 (5.1)	5 (4.5)	1
	AA + AG	172 (95.9)	66 (93.0)	0.54 (0.17–1.75)	131 (94.9)	107 (95.5)	1.14 (0.35–3.71)
rs11465802	CC	72 (40.2)	30 (42.3)	1	56 (40.6)	46 (41.1)	1
	AA + AC	107 (59.8)	41 (57.7)	0.92 (0.53–1.61)	82 (59.4)	66 (58.9)	0.98 (0.59–1.63)
rs10889677	AA	39 (21.8)	14 (19.7)	1	31 (22.5)	22 (19.6)	1
	CC + AC	140 (78.2)	57 (80.3)	1.13 (0.57–2.25)	107 (77.5)	90 (80.4)	1.19 (0.64–2.19)
CNV	CN = 2	144 (80.4)	56 (78.9)	1	109 (79.0)	91 (81.3)	1
	CN = 1, 3, 4	35 (19.6)	15 (21.1)	1.10 (0.56–2.17)	29 (21.0)	21 (18.7)	0.87 (0.46–1.62)

*OR* odds ratio, *CI* confidence interval

## Discussion

In the present study, we found that the SNP rs7518660 and the CNV in exon 11 of *IL-23R* were significantly associated with susceptibility to active pulmonary TB, meanwhile, we observed that rs11465802 was associated with drug-resistance and rs1884444 was related to cavity formation in Chinese Uygurs in China. To our knowledge, this is the first report showing that the CNV of *IL-23R* are associated with active pulmonary TB and the first association of polymorphisms in *IL-23R* with drug-sensitivity and cavitary lesion in pulmonary TB patients.

To date, the G1186A SNP in *MRC1* [29] has been reported to be associated with TB in Chinese Uygurs. There was only one study reporting the only SNP (rs11209026) of *IL-23R* associated with the development of a severe form (extensive lung infiltration) of active pulmonary TB in Tunisians [17]. Unfortunately, we couldn’t analyze the association between this polymorphism with TB, because it was excluded from further analysis due to its low minor allele frequency in the control group. The results indicate that there was ethnic difference of the SNP rs11209026 among different racial groups.

One function of IL-23 mediated by IL-23R complex is driving T cell differentiation toward Th17, which leads to an increased release of other cytokines causing inflammation, such as IL-17 and TNF [12]. The exact mechanisms how these polymorphisms regulate the function of IL-23R is currently unclear. Both the SNPs rs7518660 and rs11465802 are located in intron 7, and they may influence the expression of *IL-23R* by regulating differential splicing [30].

The signals in gene expression from SNP and CNV had little overlap, thus it is important to realize the linkage of these two polymorphisms with gene expression for understanding the mechanisms of susceptibility to TB [28]. Furthermore, both *M. tuberculosis* and *M. leprae* are intracellular bacteria, so the patients with TB and leprosy may have a similar immune response. An increase in the copy number of exon 11 in the *IL23R* gene has been reported to be associated with the paucibacillary form of leprosy [19]. Therefore, we propose a hypothesis that the CNV of *IL-23R*, if present in Chinese Uygurs, may be associated with sputum positive pulmonary TB. The result supports our hypothesis that the increase in the copy number of *IL23R* is associated with pulmonary TB in the Chinese Uygur population. Interestingly, there was weak LD between the CNV in exon 11 and rs7518660, which was significantly associated with susceptibility to pulmonary TB in Chinese Uygurs. It suggested that the CNV was an independent risk factor for pulmonary TB in contrast with SNPs among Chinese Uygurs. However, a recent study found that there was an association of *CCL3L1* copy number with TB in a northwestern Colombian population [31], but not in African and Peruvian populations [32]. All these data indicate that CNVs in some special genes may also be associated with susceptibility to TB, although the ethnic difference exists. CNV can have influence on gene expression levels by altering gene dosage, disrupting coding sequences or perturbing long-range gene regulation [33, 34]. An increase in the copy number of *IL-23R* coincided with a relatively higher fraction of memory T cells [19], but the influence of the CNV in the exon 11 of *IL23R* on its expression level needs to be further elucidated.

Among 250 clinical isolates of Mycobacterium tuberculosis, 172 isolates (68.8 %) were drug-sensitive and 78 (31.2 %) were resistant to at least one drug and 10 (4 %) were multidrug-resistant (MDR), consistent with the previous epidemiological results [22]. Interestingly, we observed that rs11465802 was significantly associated with drug-resistance, whereas it was not related to susceptibility to pulmonary TB. To date, variants in the *HLA-DRB1*, -*DQB1* [23] and *SLC11A1* [35] have shown association with *M. tuberculosis* drug sensitivity. The results demonstrate that human genetic factors are associated with the development of drug resistance of the strain in pulmonary TB patients. These data suggest that patients carrying particular gene polymorphisms may be incapable of acquiring adequate immune response to *M. tuberculosis*, and thus the infected bacteria is prone to develop drug resistance.

In addition, in this study, it was revealed that rs1884444 was associated with cavity formation, while it was unassociated with susceptibility to pulmonary TB or drug-resistance. It was reported that *SLC11A1* polymorphinisms were associated with the presence of cavitary lesions among pulmonary TB patients [35, 36]. These results suggest that human gene polymorphisms may affect their inflammatory response after the infection with *M. tuberculosis*, and thus result in the severe pathological lesion.

The IL-23/IL-17 pathway is mainly composed of IL-23, its functional receptor complex (a combination of IL-23R and IL-12Rβ1), the downstream cytokines (for example, IL-17) and other key signal molecules such as STAT4. This immune pathway and its constituent molecular members play crucial roles in autoimmune inflammatory disease pathogenesis [15]. In this study, we found the SNP rs7518660 in *IL-23R* were associated with pulmonary TB in the Chinese Uygurs. It has been reported that polymorphisms in the *IL-12 receptorβ1* gene were associated with TB in Moroccan [37] and Japanese populations [38], but not in Koreans [39]. The SNPs in *IL-17 F* were associated with pulmonary TB in Chinese Han [40] and northern Spain patients [41], but not in north Indians [42]. In addition, the polymorphisms of *STAT4* promoter-region were reported to be associated with pulmonary TB in a Moroccan population, which might impact STAT4 expression [43]. All these findings indicate that the IL-23/IL-17 pathway may be a key pathway in charging *M. tuberculosis* infection [13], although ethnic difference exists. Thus, further research on the function of the IL-23/IL-17 pathway in pulmonary TB is desired. The IL-23/IL-17 pathway was thought to be an attractive target for experimental tuberculosis therapies [44, 45]. Therefore, IL-23R, a key member in the IL-23/IL-17 pathway, also may be a potential target molecule for the therapeutic management of tuberculosis.

In our study, it was revealed that individuals with BMI < 18.5 kg/m^2^ are more likely to suffer from pulmonary TB in Chinese Uygurs. It was reported that TB incidence increased exponentially as BMI (range 18.5–30 kg/m^2^) decreased across a variety of settings with different levels of TB burden, however, the dose–response relationship was uncertain at BMI levels <18.5 kg/m^2^. There were only a few studies reporting data on TB incidence for people with BMI <18.5 kg/m^2^, but the results were inconsistent. Lonnroth K et al. [46] and Cegielski JP et al. [47] reported that people with BMI <18.5 kg/m^2^ increased TB incidence compared with those with BMI ≥18.5 kg/m^2^(including normal BMI, overweight and obesity). However, Hanrahan CF et al. [48] revealed that people with BMI <18.5 kg/m^2^ didn’t increase TB incidence compared with those with normal BMI (range 18.6-25 kg/m^2^) in HIV-infected adults.

Our study had several inherent limitations. Firstly, selection bias arose in the observation, for it was conducted using a hospital based case–control study. Secondly, the sample size was relative small, although the power of the test was over 0.8, the results were still needed to be validated in a large sample size. Additionally, because the patients were only limited to sputum positive pulmonary TB individuals, we didn’t know the association between those polymorphisms and sputum negative ones. Finally, the controls were only defined as those without LTBI. This study were conducted in TB-endemic settings, therefore we assumed that all individuals were exposed to TB. Given that the controls were uninfected, it’s unclear whether an association implies susceptibility for developing active pulmonary TB or just acquisition of LTBI.

In fact, human genetic effect on TB susceptibility is more complicated than many other diseases because of the serious confounding effects of behavioral and environmental factors [49]. However, we did not find those behavioral factors were associated with pulmonary TB, as evidenced by smoking or drinking, possibly because few participants smoke or drink due to the Muslim custom. Therefore, further study of confounding effects between IL-23/IL-17 pathway genes polymorphisms and behavioral and environmental risk factors in other ethnic groups is needed.

## Conclusions

Our study showed for the first time that *IL23R* SNPs and CNV are associated with susceptibility to pulmonary TB. Our results also revealed a significant association of *IL23R* SNPs with the severe clinical forms of pulmonary TB in the Chinese Uygur population.

## Additional files

Additional file 1: Figure S1. A. A fragment of exon11 sequenced directly by ABI 3730XL Genetic Analyzer (rs10889677, genotype AA). B. Three genotypes of rs 7518660 performed by the multiplex SNaPshot technique. C. Representative amplification plots of twofold copy number difference for *IL23R* in pulmonary TB samples compared to controls. (PDF 296 kb) Additional file 2: Table S1. Primer sets used for the initial exons sequencing of *IL23R. *(PDF 111 kb) Additional file 3: Table S2. Thirteen SNPs identified in *IL-23R* by exons resequencing from ten pairs of pulmonary TB cases and controls. (PDF 106 kb) Additional file 4: Table S3. Primer sets used for the multiplex SNaPshot of *IL23R. *(PDF 106 kb) Additional file 5: Figure S2.
*IL-23R* copy number genotyped by AccuCopy assay (Genesky Bio-Tech Co., Ltd, Shanghai, China). (PDF 161 kb) Additional file 6: Figure S3.
*IL-23R* copy number genotyped by ddPCR. A. 2-copy genotype; B. 3-copy genotype. (PDF 228 kb) Additional file 7: Table S4. LD between the CNV in *IL-23R* and rs7518660 and rs10889677 in the control group. (PDF 96 kb)
