# Supplementary material for: The variations of IL-23R are associated with susceptibility and severe clinical forms of pulmonary tuberculosis in Chinese Uygurs
Source: BMC Infect Dis. 2015 Dec 1;15:550. doi: 10.1186/s12879-015-1284-2 (PMC4665827; doi:10.1186/s12879-015-1284-2)
Supplement: Additional file 1: Figure S1. — A. A fragment of exon11 sequenced directly by ABI 3730XL Genetic Analyzer (rs10889677, genotype AA). B. Three genotypes of rs 7518660 performed by the multiplex SNaPshot technique. C. Representative amplification plots of twofold copy number difference for IL23R in pulmonary TB samples compared to controls. (PDF 296 kb) [file 12879_2015_1284_MOESM1_ESM.pdf]

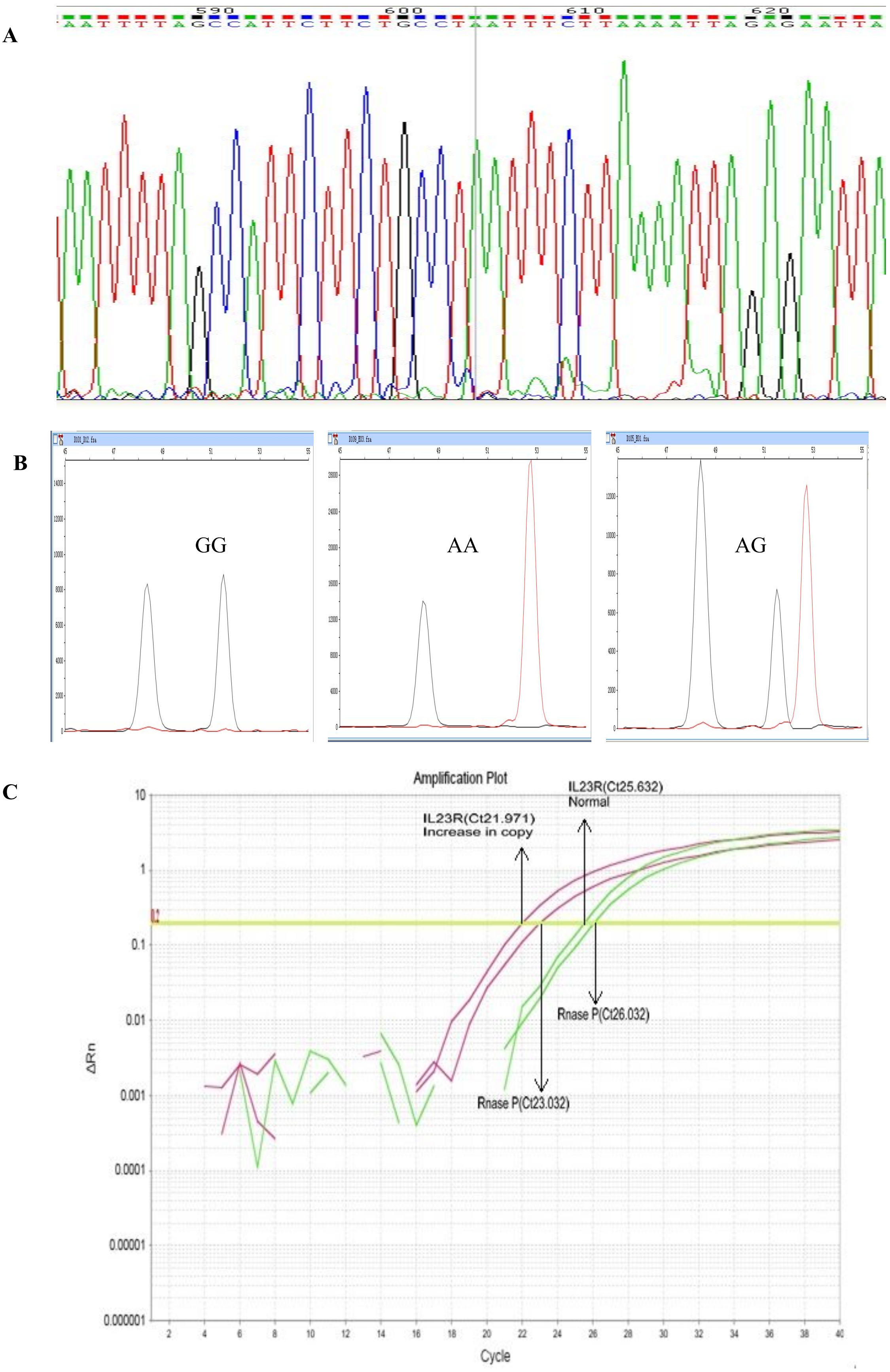

Figure S1. A. A fragment of exon11 sequenced directly by ABI 3730XL Genetic Analyzer (rs10889677, genotype AA). B. Three genotypes of rs 7518660 performed

by the multiplex SNaPshot technique. C. Representative amplification plots of twofold copy number difference for *IL23R* in pulmonary TB samples compared to controls.
