# Supplementary material for: The variations of IL-23R are associated with susceptibility and severe clinical forms of pulmonary tuberculosis in Chinese Uygurs
Source: BMC Infect Dis. 2015 Dec 1;15:550. doi: 10.1186/s12879-015-1284-2 (PMC4665827; doi:10.1186/s12879-015-1284-2)
Supplement: Additional file 2: Table S1. — Primer sets used for the initial exons sequencing of IL23R. (PDF 111 kb) [file 12879_2015_1284_MOESM2_ESM.pdf]

Table S1. Primer sets used for the initial exons sequencing of *IL23R*

Exon	Exon Length (bp)	PCR primers (sense/antisense)	PCR Products Length (bp)	Annealing Temperature
**Exon1**	56	ATAGTGACACGAGAGCCAGTCATTT	577	65℃
		CAATGCATGGCTCATCATATCGCAT		
**Exon2**	99	AACTCAGAGGAGGAGGTTTCTGATG	648	63℃
		AGGGCTGTCTAGAAGGGAAATTTGA		
**Exon3**	297	AGATACATCTAGTCCTAAAACTGT	838	55℃
		ACAAGAAGATTAAAAGAGCAACA		
**Exon4**	124	GGTGTGCCATTTGCTGAGACTGT	634	55℃
		GTAGGAAATAAGGGATAAATCAGGGTA		
**Exon5**	161	AGGCACATGCCACCAATTATTGT	539	59℃
		GCAGCACTACTCATCTCTAAGTTGTC		
**Exon6**	146	AGGATTGTAAGGGAGTGAAAGGTG	506	63℃
		AAGTCCACTGTAGCCACTGATGGTT		
**Exon7**	157	CATCTCAAAAAAAGCAGTGTGTGTT	632	63℃
		GCTCACGCAATCCTACTACCTCA		
**Exon8**	90	GCCTCATGAAATTAGTGGACACACC	563	65℃
		GCAAAATCCACCTAAGCCACTTCTT		
**Exon9**	103	GCACTTCTAAAAACCCACTGACATC	633	65℃
		AGTGATTCTGGGCTGAGGACTTAG		
**Exon10**	91	TGGCATGGTCACAGCTCACTACA	624	63℃
		CCCCCAACTCTATTGTCTCATCTCT		
**Exon11-1***	1502	TGGAGGGAGAAAGGAAAGTTGAAAG	641	63℃
		AAGCAGGGTCTGTTCTGGAATAGTT		
**Exon11-2***	1502	TCCCAGGTTACAAAAGCATCCTAAT	676	63℃
		TTCCACCTTCGGGACCTTAATTCTC		
**Exon11-3***	1502	GTGACATTTCTGTGCTCCTACCAT	745	63℃
		CACTTATTTTGTCATTTGGGGCTAT		

* Exon11 has 1502 bps, so the fragment containing exon11 is too long to be sequenced directly by ABI 3730 Genetic Analyzer. Therefore, this fragment was sliced into 3 shorter fragments. The middle short one repeated partial sequences of the other short fragments at both sides. These 3 short fragments were then amplified using a pair of primers respectively.
