# Supplementary material for: The variations of IL-23R are associated with susceptibility and severe clinical forms of pulmonary tuberculosis in Chinese Uygurs
Source: BMC Infect Dis. 2015 Dec 1;15:550. doi: 10.1186/s12879-015-1284-2 (PMC4665827; doi:10.1186/s12879-015-1284-2)
Supplement: Additional file 3: Table S2. — Thirteen SNPs identified in IL-23R by exons resequencing from ten pairs of pulmonary TB cases and controls. (PDF 106 kb) [file 12879_2015_1284_MOESM3_ESM.pdf]

Table S2. Thirteen SNPs identified in *IL-23R* by exons resequencing from ten pairs of pulmonary TB cases and controls

dbSNP ID	Position GRCh37.p13	Region	Variant	Genotype	Cases n (%)	Controls N (%)
**rs1884444**	67633812	Exon2	G/T	GG	2(0.2)	0(0.0)
				GT	4(0.4)	8(0.8)
				TT	4(0.4)	2(0.2)
**rs11465770**	67633963	Intron2	C/T	CC	8(0.8)	10(1.0)
				CT	2(0.2)	0(0.0)
**rs10889664**	67635475	Intron3	C/T	CC	5(0.5)	2(0.2)
				CT	4(0.4)	8(0.8)
				TT	1(0.1)	0(0.0)
**rs11465788**	67648294	Intron3	C/T	CC	3(0.3)	0(0.0)
				CT	4(0.4)	8(0.8)
				TT	3(0.3)	2(0.2)
**rs6687620**	67648460	Intron3	C/T	CC	8(0.8)	10(1.0)
				CT	2(0.2)	0(0.0)
**rs2863212**	67685116	Intron6	T/C	TT	9(0.9)	10(1.0)
				CT	1(0.1)	0(0.0)
**rs7530511**	67685387	Exon7	C/T	CC	8(0.8)	9(0.9)
				CT	1(0.1)	1(0.1)
				TT	1(0.1)	0(0.0)
**rs7518660**	67685443	Intron7	G/A	AA	1(0. 1)	0(0.0)
				AG	4(0.4)	7(0.7)
				GG	5(0.5)	3(0.3)
**rs11465802**	67685598	Intron7	A/C	AA	4(0.4)	1(0.1)
				AC	3(0.3)	6(0.6)
				CC	3(0.3)	3(0.3)
**rs11465804**	67702526	Intron8	T/G	TT	9(0.9)	10(1.0)
				GT	1(0.1)	0(0.0)
**rs10889671**	67705726	Intron8	G/A	AA	1(0.1)	0(0.0)
				AG	1(0.1)	1(0.1)
				GG	8(0.8)	9(0.9)
**rs11209026**	67705958	Exon9	G/A	AA	0(0.0)	0(0.0)
				AG	1(0.1)	0(0.0)
				GG	9(0.9)	10(1.0)
**rs10889677**	67725120	3'UTR	C/A	AA	3(0.3)	3(0.3)
				AC	3(0.3)	6(0.6)
				CC	4(0.4)	1(0.1)
