# Supplementary material for: The variations of IL-23R are associated with susceptibility and severe clinical forms of pulmonary tuberculosis in Chinese Uygurs
Source: BMC Infect Dis. 2015 Dec 1;15:550. doi: 10.1186/s12879-015-1284-2 (PMC4665827; doi:10.1186/s12879-015-1284-2)
Supplement: Additional file 4: Table S3. — Primer sets used for the multiplex SNaPshot of IL23R. (PDF 106 kb) [file 12879_2015_1284_MOESM4_ESM.pdf]

Table S3. Primer sets used for the multiplex SNaPshot of *IL23R*

**dbSNP ID**	**Variant**	**PCR primers(sense/antisense)/ extension primers**	**Length(bp)** *
**rs1884444**	G/T	TCCCTAATCAAAGGTTCCCATCAAA	181bp
		GGGCTATTACTGCATCCCATTGA	
		TTTTTTTTTTTTTTTTTTTTTTTTTTTTCCTGCTTCCAGACATGAATCA	
**rs11465770**	C/T	GCTGGTGTCATGGAGGTATGGTGT	191bp
		TGGAAGGAGATTGGGCCATTTTTA	
		TTTTTTTTACATTTTTTCTAGTCTAATAATATTTGTATTCTTCA	
**rs11465788**	C/T	GCCTCCTTGAGCTGCAGTGGG	754bp
		CGAAGTTCTCTGGTCCCAAGCCA	
		GCAATGCCTCGCCCTGCTT	
**rs6687620**	C/T	CCCACTGTCTGATAAACCCCAGTG	191bp
		ATCTGGCGGATCTGCAACAAAA	
		TTTTTTTTTTTCCTATTCAGCCATCTTGGCT	
**rs7530511**	C/T	CATCTCAAAAAAAGCAGTGTGTGTT	632bp
		GCTCACGCAATCCTACTACCTCA	
		TTTTTTTCCTGTTTCAGGTGTTTTATGAAAAAAC	
**rs11465802**	A/C	GGTACTGGCAGCCTTGGAGTTC	267bp
		AATGCATTCTACCACCCAGGCTAA	
		TTTTTTTTTTTTTTTTTTTTTTTTTTTTGAGACCCTAGTCTGTACAGAAAAATAAT	
**rs11465804**	T/G	CCCAGGTCACATCAAAAGCATTC	291bp
		AAAGCCGGCAAAATCCACCTAA	
		TTTTTTTTTTTTTTTTTTTTTTTTTTTTTGATGGGTTAAAATGGGCAATT	
**rs10889671**	G/A	CCGACCACATTTGCCAGAGATG	160bp
		TGGGGGGAAGCAAAATTAACCT	
		TTTTTTTTTTTTTTTTTTGGGGGAAGCAAAATTAACCTTTAC	
**rs11209026**	G/A	TGGGAATGATCGTCTTTGCTGTT	294bp
		GGAAGCTTTTCTGGCAGGGTCA	
		TTTTTTTTTTTTTTTTTCTGCAAAAACCTACCCAGTT	
**rs10889677**	C/A	TCTGTGCTCCTACCATCACCATGT	160bp
		TGACCATGAAGCATGTTCCACCT	
		TTTTTTTTTTTTTTTTTTTTTTTTCGGGACCTTAATTCTCTAATTTTAAGAAAT	
**rs2863212**	T/C	GGCTGAACTGGGGTGGAACTGC	470bp
		AGGCTGCCAGTACCTTTTGCCT	
		TTTTTTTCTCTAGGAAAACAAAGTGTTCTTTATATA	

		AAAC	
**rs7518660**	G/A	GGTACTGGCAGCCTTGGAGTTC	122bp
		GAGCCACTGTGCTCAGCAGAAA	
		TTTTTTTTTTTTTTTTTTTTTCAGCAGAAAAGATATAT AAAGAAAAGCC	
**rs10889664**	C/T	TGCACTGCTGAATGTCCCAAAC	289bp
		TAGGAATACCGGGCACCTGTGA	
		TTGCTACAGTTCATATAGTTCACCAACCT	

* The length of the PCR products.
