# Supplementary figures and images for: The variations of IL-23R are associated with susceptibility and severe clinical forms of pulmonary tuberculosis in Chinese Uygurs
Source: BMC Infect Dis. 2015 Dec 1;15:550. doi: 10.1186/s12879-015-1284-2 (PMC4665827; doi:10.1186/s12879-015-1284-2)

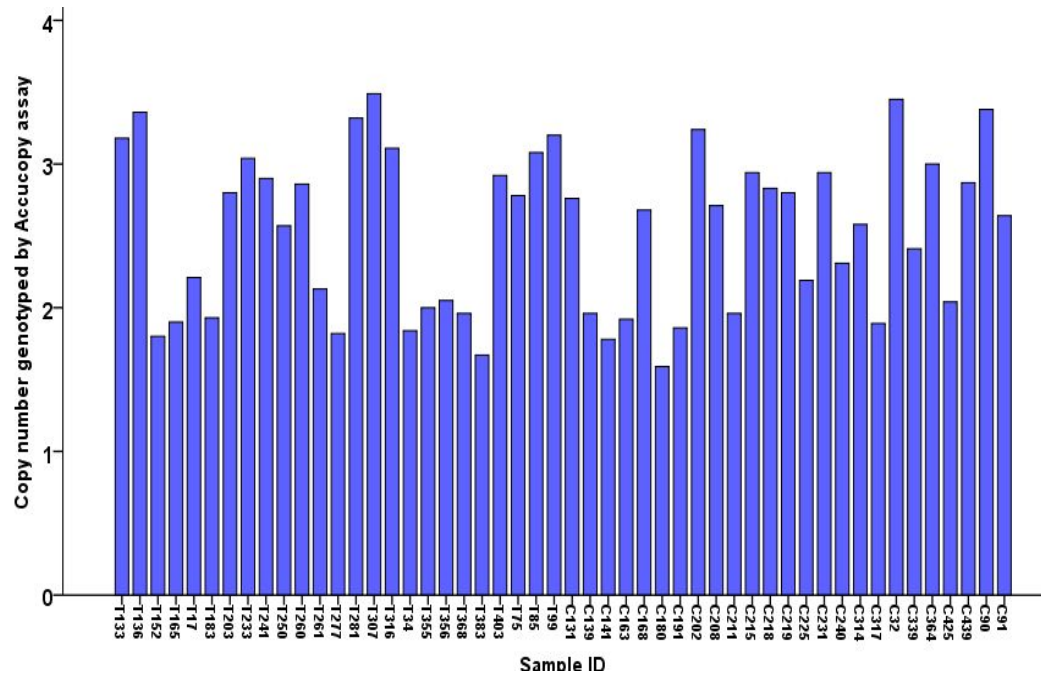

**Figure S2.** *IL-23R* copy number genotyped by AccuCopy assay (Genesky Bio-Tech Co., Ltd, Shanghai, China).

Supplement: Additional file 5: Figure S2. — IL-23R copy number genotyped by AccuCopy assay (Genesky Bio-Tech Co., Ltd, Shanghai, China). (PDF 161 kb) [file 12879_2015_1284_MOESM5_ESM.pdf]

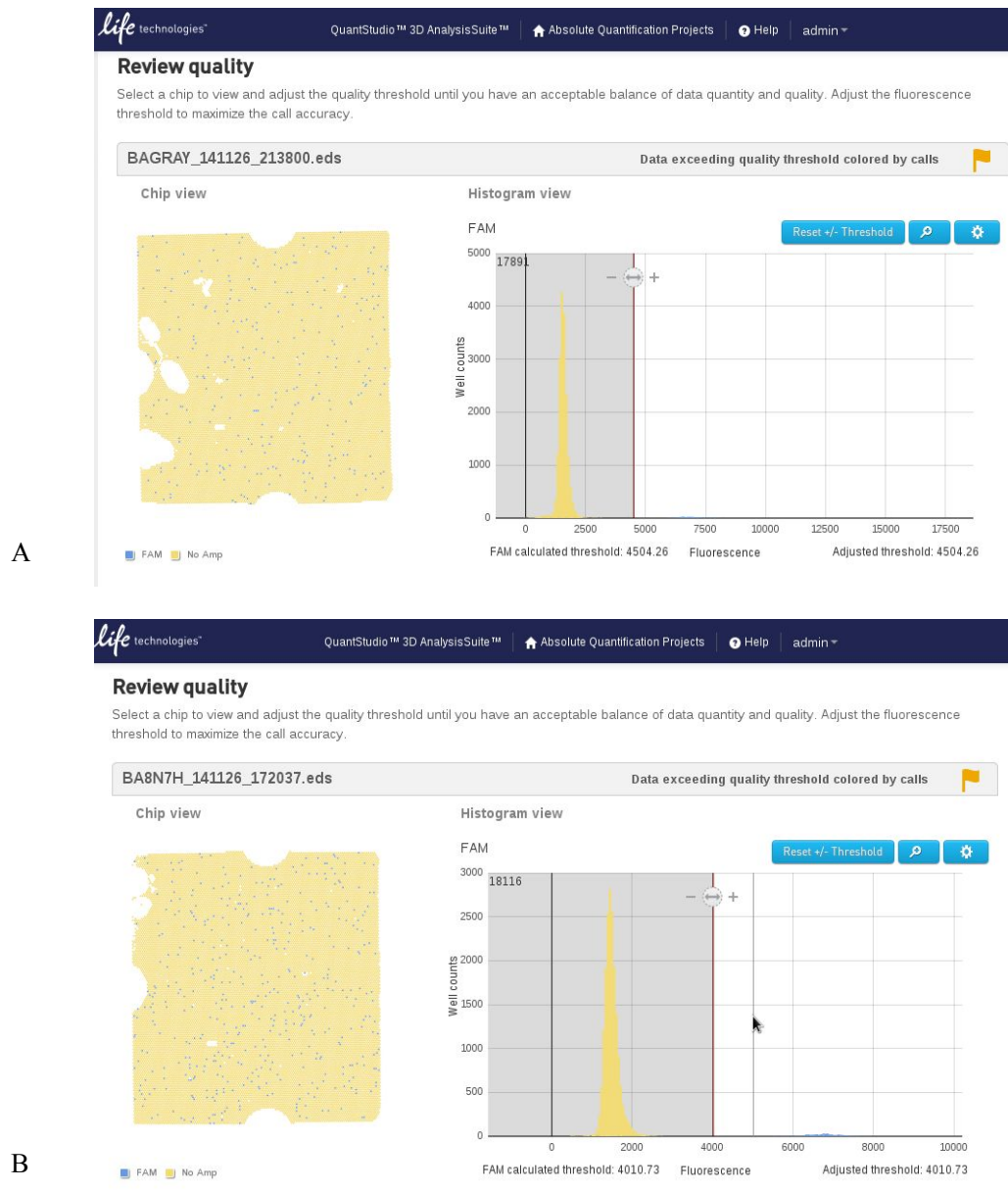

**Figure S3.** *IL-23R* copy number genotyped by ddPCR.

A. 2-copy genotype; B. 3-copy genotype.

Supplement: Additional file 6: Figure S3. — IL-23R copy number genotyped by ddPCR. A. 2-copy genotype; B. 3-copy genotype. (PDF 228 kb) [file 12879_2015_1284_MOESM6_ESM.pdf]
