# Supplementary material for: The variations of IL-23R are associated with susceptibility and severe clinical forms of pulmonary tuberculosis in Chinese Uygurs
Source: BMC Infect Dis. 2015 Dec 1;15:550. doi: 10.1186/s12879-015-1284-2 (PMC4665827; doi:10.1186/s12879-015-1284-2)
Supplement: Additional file 7: Table S4. — LD between the CNV in IL-23R and rs7518660 and rs10889677 in the control group. (PDF 96 kb) [file 12879_2015_1284_MOESM7_ESM.pdf]

Table S4. LD between the CNV in *IL-23R* and rs7518660 and rs10889677 in the control group

SNP	CNV Haplotype	r^2	D'
rs7518660	HCN 0	0.003	1.000
	HCN 1	0.001	0.123
	HCN 2	0.005	0.264
rs10889677	HCN 0	0.005	1.000
	HCN 1	0.001	0.157
	HCN 2	0.005	0.339

CNV, copy number variation; CN, copy number.

HCN 0, LD measured between diallelic SNP and phased haplotype copy number of deletion vs duplication/normal; HCN 2, LD measured between SNP and duplication vs deletion/normal. *p* value determined by covariance of SNP and CNV genotypes. For D' and r^2, LD measures derived from phased haplotypes. Due to the very low MAF of HCN 0 (0.06), the D' value was 1, which didn't mean very strong LD between HCN0 and rs7518660 and rs10889677, because the corresponding r^2 was lower than 0.01, which was not affected by the minor distribution frequency of the haplotypes, meaning the very weak LD.
